# Interaction between early-life pet exposure and methylation pattern of *ADAM33* on allergic rhinitis among children aged 3–6 years in China

**DOI:** 10.1186/s13223-021-00526-5

**Published:** 2021-05-01

**Authors:** Yu Zhang, Meiyu Tan, Xiaoqiong Qian, Cong Li, Lei Yue, Yuehong Liu, Song Shi

**Affiliations:** 1 Department of Otorhinolaryngology, Tongren Hospital, Shanghai Jiao Tong University School of Medicine, Shanghai, 200336 China; 2 Department of Laboratory Diagnosis, Tongren Hospital, Shanghai Jiao Tong University School of Medicine, Shanghai, 200336 China

**Keywords:** Allergic rhinitis, Methylation, *ADAM33*, Pet exposure

## Abstract

**Background:**

Recent research has pointed out the important roles of epigenetic modifications in the development and persistence of allergic rhinitis (AR), especially in relation to DNA methylation of disease-associated genes. We investigated whether AR susceptibility genes were epigenetically regulated, and whether methylation modulation of these genes in response to early-life environment could be a molecular mechanism underlying the risk for AR onset in a cohort of children aged 3–6 years in China.

**Methods:**

Peripheral blood mononuclear cell (PBMC) samples were collected from 130 children patients, aged 3–6 years and diagnosed with AR; and 154 matched controls to detect promoter methylation in 25 AR susceptibility genes with the MethylTarget approach. Methylation levels were compared for each CpG site, each amplified region, and each gene. In addition, the relationship among DNA methylation, early-life environmental risk factors and AR onset were assessed.

**Results:**

Maternal allergic history (P = 0.0390) and pet exposure (P = 0.0339) were significantly associated with increased AR risk. Differential methylation analyses were successfully performed for 507 CpG sites, 34 amplified regions and 17 genes and significant hypomethylation was observed in the promoter region of *ADAM33* in AR patients [multiple test-corrected (FDR) P-value < 0.05]. Spearman correlation analysis revealed that the hypomethylation of *ADAM33* was significantly associated with higher eosinophil counts (Spearman’s ρ: − 0.187, P-value = 0.037). According to the results of the multiple regression analysis, after adjusting for cofounders, the interaction of early-life pet exposure with methylation level of *ADAM33* increased the risk for AR onset 1.423 times more in children (95% CI = 0.0290–4.109, P-value = 0.005).

**Conclusion:**

This study provides evidence that early-life pet exposure and low methylation level of *ADAM33* increase AR risk in children, and the interaction between pet exposure and methylation level of *ADAM33* may play an important role in the development of AR.

## Background

Allergic rhinitis (AR) is a common IgE-mediated disorder involving troublesome symptoms of nasal congestion, nasal itch, sneezing, and associated eye symptoms [1]. AR is a multifactorial disease triggered by genetic and environmental factors as well as their interaction. Classical genetic association studies including genome-wide association studies (GWASs) were unable to explain the missing heritability and such a highly increasing prevalence of AR [2–5].

Considering the dramatic increase in the prevalence of AR [6], the epigenetic modification may be an important genetic factor to better understand the environmental effects on allergic diseases. DNA methylation, which refers to the addition of a methyl group to DNA, plays a crucial role in controlling the gene expression patterns. Recent genome-wide DNA methylation profiling studies in T-cells have shown clearly and robustly distinguished methylation profiles in AR patients from controls [7]. Zhang et al. have modeled differences between genome-wide DNA methylation and allergic sensitization during adolescence. It has been found that DNA methylation at cg10159529 is associated with AR and strongly correlated with expression of IL5RA [8]. Methylation modulation of several candidate genes has also been reported to have an important role in AR development [9].

Like many chronic health conditions, AR is complex and stems from complex gene–environment interactions [10]. Recent studies have supported a relationship between external exposome in the prenatal and early-life risk factors and their effects on the development of allergic diseases later in life [11]. The association of these risk factors and the subsequent development of AR focuses on maternal allergic history [12], mode of delivery [13], microbial exposure [14, 15], indoor allergens (furred pet exposure, for example) [16], and environmental air pollutants [17] during early-life have been previously reported. One mechanism underlying the effect of air pollutants on AR using mice model has been reported recently showing that PM2.5 exposure exacerbates AR by increasing DNA methylation in the IFN‐gamma gene promoter in T cells [18].

Considering the established roles of DNA methylation and important effects of the early-life environment on AR development, we conducted a cross-sectional study to explore the association between early-life environment risk factors, methylation of AR susceptibility genes and AR risk in a population of Chinese children.

## Methods

### Subjects and DNA specimens

This study was approved by the Ethics Committee of Tongren Hospital Affiliated to Shanghai JiaoTong University, School of Medicine (NO: TR2019.050.01). Written informed consent was obtained from all legal guardians of the participating childrens prior to blood collection. A total of 130 subjects with AR were recruited from the Department of Otorhinolaryngology. The control population included 154 healthy children who underwent a regular physical examination in the same ear, nose and throat (ENT) clinic as the AR patients. Individuals with history of asthma or atopic dermatitis were excluded. All subjects were born in and permanent residents of Shanghai. Genomic DNA extraction was performed on PBMC samples collected and isolated using the QIAamp DNA Blood kit (QIAGEN, Germany), according to the manufacturer’s instructions. Venous whole-blood eosinophil counts were performed using an XN-9000 (Sysmex Co., Kobe, Japan).

### Clinical diagnoses

According to the Initiative on Allergic Rhinitis and its Impact on Asthma guidelines, a thorough history including typical AR symptoms, physical examinations and an allergen skin prick test (SPT) was used to establish the diagnosis of AR [19]. SPT was performed by trained practitioners and positivity was defined as described elsewhere [20]. The recruited pediatric patients were carrying classic AR symptoms and positive SPT. While patients with comorbid asthma condition were excluded by lung function and bronchial provocation testing.

### Questionnaire survey

A questionnaire was answered by mothers of all of the study subjects, and the following variables were recorded: gender, weight, height, history of maternal allergic disease, season of birth (March to August was defined as spring–summer; September to February was defined as autumn–winter), secondhand smoke exposure (yes or no), and pet exposure (yes or no) in the child’s home before their kindergarten life.

### Selection of AR-associated genes

Top twenty-five AR-susceptibility genes were selected using Phenopedia database (https://phgkb.cdc.gov/PHGKB/startPagePhenoPedia.action). Genes were ranked according to the number of previously published gene-disease association studies, thus providing a disease-centered view of genes involved in AR [21].

### DNA methylation analysis

DNA methylation level was analyzed by a multiplex PCR and next-generation sequencing-based targeted CpG methylation analysis method—MethylTarget™ (Genesky Biotechnologies Inc., Shanghai, China). The validity and reliability of this method have been previously reported [22–24]. Specifically, CpG islands located in the promoter of genes of interest were selected according to the following criteria: (1) 200 bp minimum length; (2) above 50% GC-content; (3) above 0.6 ratio of observed/expected CpG. Sodium bisulfite conversion of DNA was performed using EZ DNA Methylation™-GOLD Kit (Zymo Research), following the manufacturer’s protocols. Primers were designed and provided by Genesky Company for multiplex PCR amplification using HotStarTaq DNA polymerase kit (TAKARA, Tokyo, Japan). After PCR amplification and library construction, samples were sequenced (Illumina MiSeq Benchtop Sequencer, CA, USA) using the paired-end sequencing protocol according to the manufacturer’s guidelines (Additional file 1).

One gene on X-chromosome and seven genes without CpG islands or failed to be amplified were excluded from the following analysis. In total, 34 amplicons of CpG regions in the promoter of 17 genes were sequenced (the detailed information related with gene names, location of the amplicons, amplification primers, and product size can be found in Additional file 2. All samples achieved a mean coverage of > 800 × and no significant difference in bisulfite conversion efficiency was identified between the groups (Additional file 3). Methylation level at each CpG site was calculated as the percentage of the methylated cytosines over the total tested cytosines. The average methylation level of all measured CpG sites within the amplified region or the gene was used for identifying differentially methylated amplicons and genes.

### Statistical analysis

The data were analyzed using SPSS version 18.0 software (SPSS Inc., Chicago, IL, USA). For basic characteristics and potential risk factors, the differences between the groups were measured using the χ^2^ test for categorical variables or t-test for continuous variables. Mann–Whitney *U* test was used to compare methylation levels of the AR-associated genes between AR patients and control subjects. Spearman correlation test was used to evaluate the relationship among study variables in AR patients. Receiver operating characteristic (ROC) curve and area under curve (AUC) were used to evaluate the predictive power or feasibility of the methylation as a biomarker for AR. False discovery rate (FDR) was applied for the multiple test correction. Associations were considered significant when P values were less than 0.05.

## Results

### Demographic data and clinical manifestations

A total of 130 patients with AR (78 boys, 52 girls) and 154 controls (98 boys, 56 girls) were recruited. No statistically significant differences were found between cases and controls in terms of sex, age, weight, and height (all P-value > 0.05). Maternal allergic history (P = 0.0390) and pet exposure (P = 0.0339) significantly increased the risk of developing AR. However, no effects were found on season of birth or exposure to second-hand smoke for AR risk. The demographic details of the sample are given in Table 1.

**Table 1:** Characteristics of study participants

Variable	AR	Control	P-value
N	130	154	
Male (%)	78 (60.00)	98 (63.64)	0.532
Age (SD), month	53.72 (11.04)	52.14 (10.11)	0.209
Weight (SD), kg	19.27 (1.76)	18.25 (1.77)	0.0800
Height (SD), mm	105.81 (4.98)	106.09 (5.07)	0.581
Maternal allergic history (%)	75 (57.69)	70 (45.45)	*0.0390*
Season of birth			
Spring–summer	62	77	0.698
Autumn–winter	68	77	
Exposed to pet (%)	49 (37.69)	40 (25.97)	*0.0339*
Exposed to second-hand smoke (%)	59 (45.38)	66 (42.86)	0.669
Venous whole-blood eosinophil counts (cells/mL^3^)	372.35 (108.02)		

### Differentially methylated sites, amplicons, and genes

**Fig. 1:**
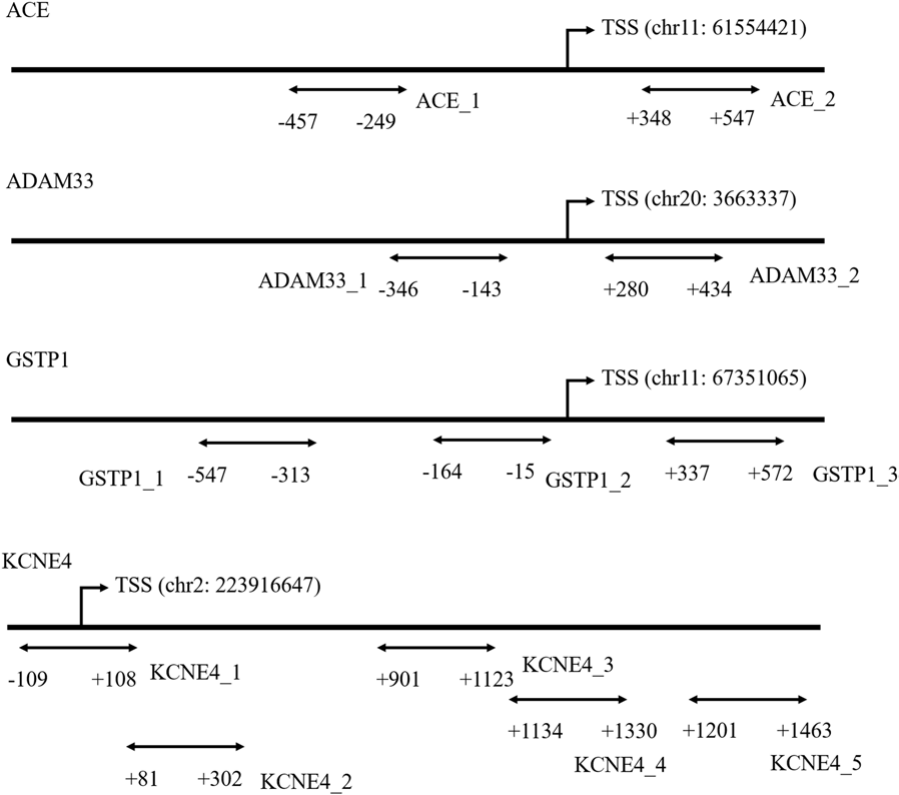
CpG regions sequenced around promoter of *ACE, ADAM33, GSTP1* and *KCNE4*. Short lines with arrows indicate amplicons of CpG region analyzed in this study, all of which are located in CpG islands around gene promoters. Range of each region is indicated by its relative distance (in bp) to TSS

The results showed that 34 amplicons contained 507 CpG sites in promoter region of the 17 AR-susceptibility genes sequenced (one to three amplicons for each gene, detailed information can be found in Additional file 2. To better characterize the DNA methylation of the 17 AR-susceptibility genes, differential methylation analyses were performed for the 507 CpG sites, 34 regions and 17 genes, respectively. The results showed that 55 of 507 CpG sites, all located on either gene *ACE* or *ADAM33*, were differently methylated in AR patients compared to controls (all P < 0.05) (Additional file 4). However, the CpG site at the position of 24 bp of the first amplicon of *ACE* (ACE_1) (Fig. 1) was the only CpG site remained significant after correcting for multiple testing (FDR P = 0.0337).

As shown in Table 2, three amplicons carrying the CpG region of the genes *GSTP1*, *ADAM33* and *KCNE4* (GSTP1_1, ADAM33_1 and KCNE4_3) were differently methylated in AR patients compared to controls (all P < 0.05). Location of each amplicon is shown in Fig. 1. The difference between GSTP1_1 and ADAM33_1 was still significant after correcting for multiple testing (FDR P = 0.04833). In addition, we evaluated the differences between AR cases and controls at the genomic DNA methylation levels. The results exhibited that the DNA methylation levels of *ADAM33* and *GSTP1* genes were significantly different between AR patients and controls (all P < 0.05). The differences were still significant after correcting for multiple testing (P = 0.0483) (Table 3). Since there were no significant methylation differences for all the CpG sites in *GSTP1*, *ADAM33* was selected for following analysis. The methylation levels of promoter regions in *ADAM33* of AR and control groups are shown in Fig. 1.

**Table 2:** Differentially methylated amplicons of CpG region between AR samples and control samples

Target/gene	Mean in AR	Mean in control	MethylDiff	P-value (U test)	FDR P-value (U test)
GSTP1_1	0.30953	0.31456	− 0.00502	*0.00142*	*0.0483*
ADAM33_1	0.37788	0.38639	− 0.00852	*0.00261*	*0.0483*
KCNE4_3	0.91735	0.90976	0.00759	*0.04671*	0.3729

Target, the name of the amplicon; mean in AR, average methylation degree of the AR group; mean in control, average methylation degree of the control group; MethylDiff, average methylation degree of the AR group minus average methylation degree of the control group; P value (U-test): the U-test model is used to calculate the P value

**Table 3:** Differentially methylated genes between AR samples and control samples

Gene	Mean in AR	Mean in control	MethylDiff	P-value (U-test)	FDR P-value (U-test)
ADAM33	0.20249	0.20783	− 0.00534	*0.00243*	*0.0483*
GSTP1	0.10541	0.10323	0.00218	*0.00424*	*0.0483*

Mean in AR, average methylation degree of the AR group; mean in control, average methylation degree of the control group; MethylDiff, average methylation degree of the AR group minus average methylation degree of the control group; P value (U-test): the U-test model is used to calculate the P value

### Differentially methylated CpG sites in *ADAM33*

**Fig. 2:**
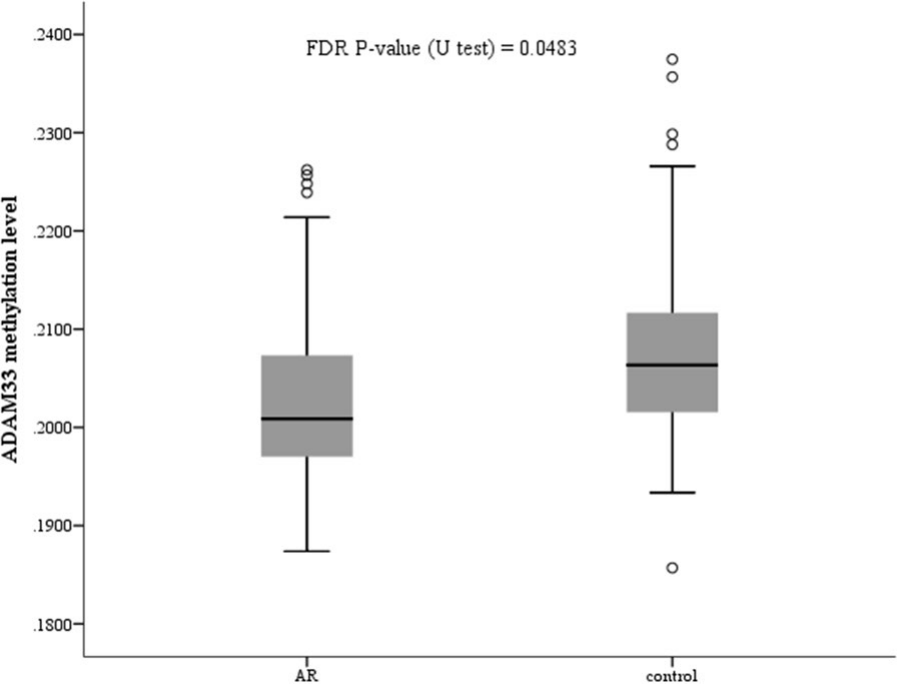
The methylation levels of promoter region in *ADAM33* gene in PBMC of paired AR samples and control samples

**Fig. 3:**
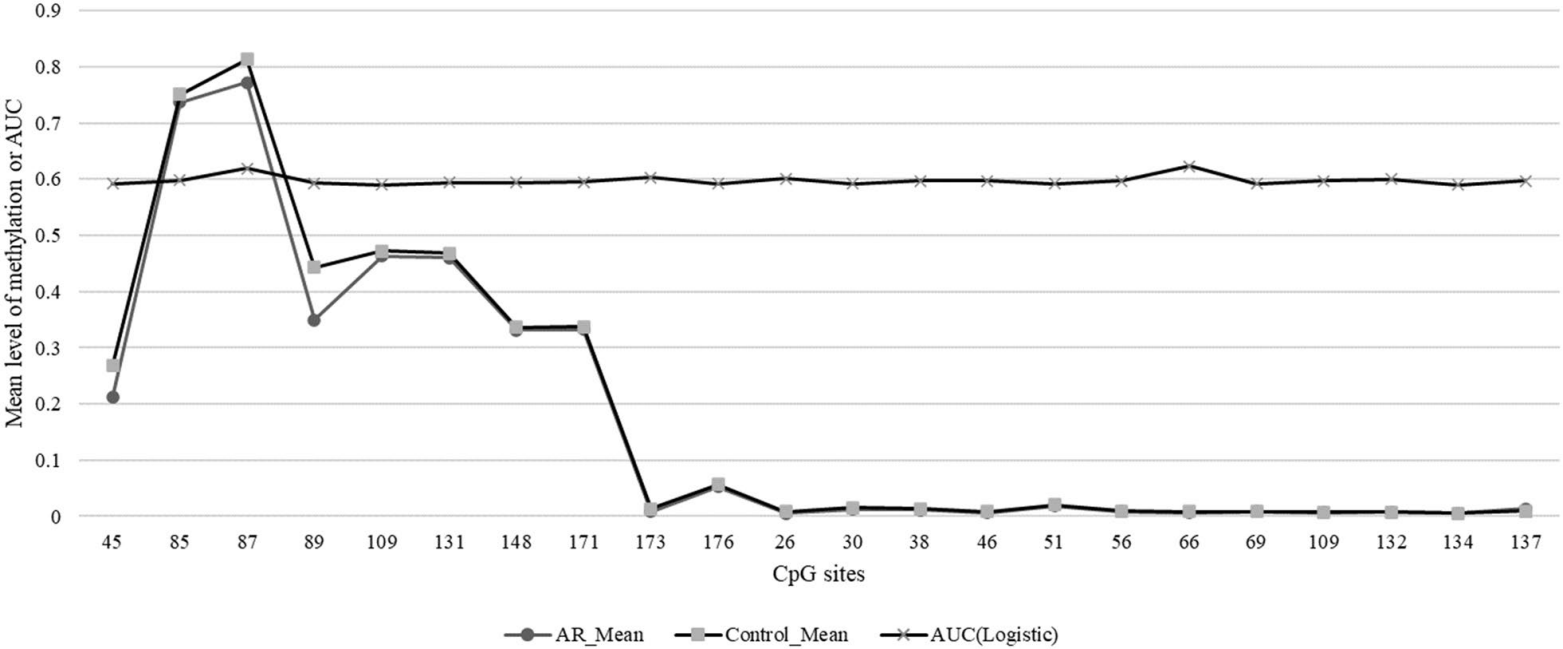
The methylation levels of each CpG site in *ADAM33* gene in PBMC of paired AR samples and control samples, and AUC value of each CpG site in *ADAM33* gene showing the potentiality of the CpG site as a biomarker for AR

To evaluate the potentiality of the CpG sites as a biomarker for AR, receiver operating characteristic curve (ROC) analysis was performed on all the CpG sites in *ADAM33* gene (Fig. 2). Mean methylation level and the AUC of ROC curve of each CpG site are shown in Fig. 3 and Table 4. The highest AUC was 0.6233 for the CpG site at position 66 bp of the sequencing region of ADAM33_2. Four CpG sites (CpG sites at positions of 45 bp, 85 bp, 87 bp and 89 bp of ADAM33_1) had mean methylation level difference above 0.01 in AR and controls.

**Table 4:** Methylation sites in promoter region of *ADAM33*

Target	Position	Type	P-value (Utest)	FDR P-value (Utest)	OR(L95-U95) (logistic)	AUC (logistic)	MethylDiff	Mean in AR	Mean in control
ADAM33_1	45	CG	0.052	0.475	0.9761 (0.9549–0.9978)	0.592	− 0.05618	0.21179	0.26797
ADAM33_1	85	CG	*0.040*	0.456	0.9567 (0.9024–1.0143)	0.598	− 0.01416	0.73703	0.75119
ADAM33_1	87	CG	0.055	0.475	1.0328 (0.995–1.072)	0.619	− 0.04202	0.77197	0.81399
ADAM33_1	89	CG	*0.049*	0.475	1.009 (0.9988–1.0193)	0.593	− 0.09300	0.35000	0.44300
ADAM33_1	109	CG	0.057	0.475	0.8988 (0.8054–1.003)	0.590	− 0.00904	0.46346	0.47250
ADAM33_1	131	CG	*0.047*	0.462	0.9215(0.8309–1.022)	0.594	− 0.00761	0.46077	0.46838
ADAM33_1	148	CG	*0.046*	0.462	0.9051 (0.7932–1.0328)	0.594	− 0.00564	0.33127	0.33692
ADAM33_1	171	CG	*0.044*	0.459	0.9589 (0.8777–1.0477)	0.595	− 0.00518	0.33279	0.33797
ADAM33_1	173	CG	*0.042*	0.459	0.6178 (0.4049–0.9426)	0.603	− 0.00498	0.00858	0.01357
ADAM33_1	176	CG	0.053	0.475	0.8126 (0.6548–1.0083)	0.592	− 0.00456	0.05248	0.05704
ADAM33_2	26	CG	*0.045*	0.460	0.5744 (0.3513–0.9392)	0.601	− 0.00304	0.00577	0.00881
ADAM33_2	30	CG	0.051	0.475	0.5615 (0.3543–0.8898)	0.593	− 0.00301	0.01210	0.01512
ADAM33_2	38	CG	*0.040*	0.456	0.7737 (0.5536–1.0813)	0.597	− 0.00231	0.01141	0.01372
ADAM33_2	46	CG	*0.044*	0.459	0.4853 (0.243–0.9691)	0.598	− 0.00222	0.00628	0.00851
ADAM33_2	51	CG	0.053	0.475	0.5236 (0.2937–0.9335)	0.592	− 0.00207	0.01878	0.02086
ADAM33_2	56	CG	*0.039*	0.456	0.7242 (0.4734–1.108)	0.597	− 0.00181	0.00791	0.00972
ADAM33_2	66	CG	0.058	0.475	0.1676 (0.0344–0.8158)	0.623	− 0.00162	0.00684	0.00846
ADAM33_2	69	CG	0.053	0.475	0.8777 (0.6093–1.2642)	0.592	− 0.00098	0.00792	0.00889
ADAM33_2	109	CG	*0.041*	0.458	0.122 (0.0229–0.6488)	0.597	− 0.00087	0.00635	0.00721
ADAM33_2	132	CG	*0.038*	0.456	0.7905(0.4372–1.4293)	0.601	− 0.00071	0.00693	0.00764
ADAM33_2	134	CG	0.058	0.475	0.4438 (0.1491–1.3204)	0.590	− 0.00070	0.00511	0.00582
ADAM33_2	137	CG	0.055	0.475	1.5811 (1.081–2.3123)	0.597	0.00475	0.01382	0.00908

Target, the name of the amplicon; POS, the specific location of the methylation site in the amplicon; P value (U-test): the U-test model is used to calculate the P value; OR (L95–U95) (logistic), AUC (logistic), odds ratio and area under curve was calculated through the logistic regression model; mean in AR, average methylation degree of the AR group; mean in control, average methylation degree of the control group; MethylDiff, average methylation degree of the AR group minus average methylation degree of the control group

### Correlation of *ADAM33* methylation with clinical manifestations

**Fig. 4:**
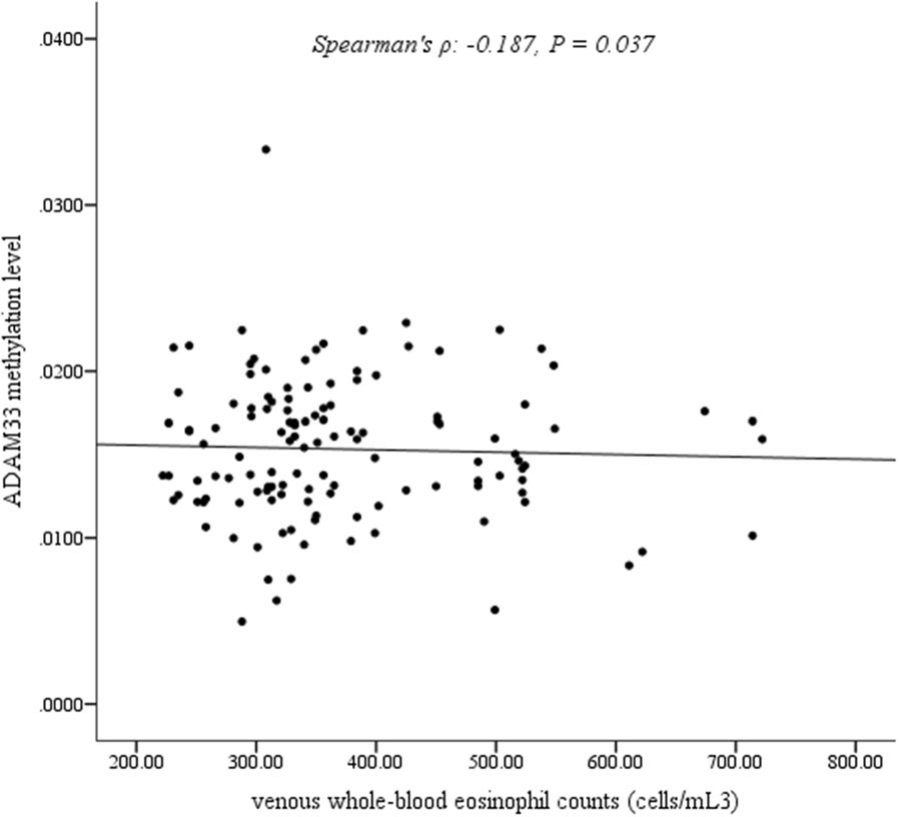
Correlation of the mean methylation status in the promoter CpG islands of *ADAM33* with eosinophil counts (Spearman’s rank correlation)

The venous whole-blood eosinophil count (normal range: 50–500 cells/mL^3^) was 372.35 ± 108.02 cells/mL^3^ in AR patients. Spearman correlation analysis revealed that the hypermethylation of *ADAM33* was significantly associated with lower eosinophil count (Spearman’s *ρ*: − 0.187, *P* = 0.037; Fig. 4). Moreover, the methylation level of the CpG site at the position of 24 bp of the first amplicon of *ACE* (ACE_1) was not significantly associated with the total eosinophil count (Spearman’s *ρ*: − 0.022, *P* = 0.808) (Additional file 5).

### *ADAM33* promoter methylation level as a risk factor in AR

We compared the mean *ADAM33* promoter methylation levels between the AR and control groups stratified by maternal allergic history and exposure to pet, the two risk factors identified in this study (Table 5). In the AR group, children with daily life pet exposure had significantly lower methylation levels compared to those without pet exposure (P-value = 0.009). The difference in control group was not significant. Impact of maternal allergic history on methylation level of *ADAM33* was not found in both AR and control groups.

**Table 5:** *ADAM33* promoter methylation levels between the AR and control groups stratified by environmental risk factors

Variable	AR	Mean *ADAM33* promoter methylation levels	Control	Mean *ADAM33* promoter methylation levels
N	130		154	
Maternal allergic history				
Yes	75 (57.69)	0.2018 ± 0.008417	70 (45.45)	0.2073 ± 0.01042
No	55 (42.31)	0.2029 ± 0.007894	84 (54.55)	0.2081 ± 0.01130
P-value		0.346		0.897
Exposed to pet				
Yes	49 (37.69)	0.2012 ± 0.008346	40 (25.97)	0.2098 ± 0.01172
No	81 (62.31)	0.2044 ± 0.007349	114 (74.03)	0.2062 ± 0.01015
P-value		*0.009*		0.290

Values are presented as means ± SD or numbers. A comparison between AR and control groups by Mann–Whitney U test. Significant results are in italics

Based on the results of the multiple regression analyses, adjusted for gender, age, height, weight, season of birth and exposure to second-hand smoke, pet exposure was significantly related to higher risk of developing AR. Furthermore, the interaction between pet exposure and methylation level of *ADAM33* was significantly related to AR risk (OR = 1.423, 95% CI = 0.0290–4.109, P-value = 0.005) (Table 6).

**Table 6:** Multiple regression analysis for analyzing the relationship among risk factors, the methylation level of *ADAM33* and AR, AR as the dependent variable

Risk factor	β	OR (95% CI)	P-value
Maternal allergic history^a^	0.561	1.752 (0.817–3.761)	0.150
Exposed to pet^a^	0.721	2.057 (1.029–4.109)	*0.001*
Methylation level^b^	− 0.225	0.799 (0.645–1.006)	0.114
Methylation level × exposed to pet	0.353	1.423 (1.007–1.639)	*0.005*
Methylation level × maternal allergic history	− 0.212	0.809 (0.699–1.105)	0.169

Adjusted for gender, age, height, weight, season of birth and exposure to second-hand smoke

*OR* odds ratio, *CI* confidence interval

^a^0 = without, 1 = with

^b^Methylation levels were rescaled to rank

## Discussion

The aim of the study was to investigate the relationship among environmental risk factors, the methylation level of AR candidate genes reported from polymorphism association studies and AR risk in a cohort of children aged 3–6 years in China. We found that among the 17 investigated genes, the DNA methylation level of *ADAM33* was significantly lower in the AR group than controls and the difference was still significant after correcting for multiple testing. Furthermore, we showed that pet exposure was related to the higher risk for AR with respect to DNA methylation level in promoter region of *ADAM33*.

In our study, maternal allergic history was a strong risk factor for AR among a cohort of Chinese children. This result is consistent with that of a previous study involving a cohort of 6 year-old children, reporting that maternal allergic history was associated with higher risk for AR development [25]. The biological mechanism proposed was that childhood allergy development was impaired by maternal allergic disease history through impairment of neonatal regulatory T-cells [12]. Furthermore, plenty of contradictory associations exist as whether furred pet exposure (cats and dogs) may be a risk or a protective factor for the AR development [16, 26, 27]. We also found that pet exposure was another risk factor for AR, which is consistent with a recent study from Finland showing that dog and cat exposure in early life could increase the risk of developing pet allergies [28]. However, the cumulative evidence from several systematic reviews suggest that pet allergen exposure has not increased the risk for developing allergic disease [16, 29, 30]. The discrepancies are likely due to the ubiquitous nature of pet allergens, while pet owners are more concerned about sanitation and many other hygiene-related reasons.

Genetic association studies have advanced our understanding of genetic risk factors for allergic diseases. In the latest GWAS of AR, 41 AR-related risk loci have been reported, including 20 loci that had not previously been related to the disease [2–5], however, none of them have been confirmed to be a hub gene in the development or persistence of allergic diseases. In this study, 17 candidate genes for association with AR were identified using Human Genome Epidemiology (HuGE) Navigator [21] and methylation levels of promoter regions were compared in PBMCs of AR cases and control individuals.

One CpG site in the promoter region of *ACE* was found as the only CpG site remained significant after correcting for multiple testing in our study. Although methylation level of this CpG site was not significantly associated with the total eosinophil count in the case group, however, methylation of *ACE* might still play an important role in the development of AR, considering transcription of the mRNA might be regulated by DNA methylation at one specific site in its promoter [31].

Disintegrin and metalloproteinase 33 (ADAM33), the first asthma-susceptible gene identified by positional cloning, was the only gene identified with significant methylation level differences between the groups at the CpG site level, amplicon level and gene level. Notably, *ADAM33* has been extensively reported as a susceptibility gene in bronchial hyperresponsiveness, asthma and AR [21, 32–34]. *ADAM33* is expressed in the smooth muscle, myofibroblasts, and fibroblasts of asthmatic airways, thus the function of this protein might be involved in the airway remodeling [35]. Various lines of evidence from previous human and animal studies have indicated that the expression level of *ADAM33* was upregulated during acute or chronic lung inflammation [36]. Even though the existence of this functional link between *ADAM33* and allergic airway inflammation, its role in the pathophysiology of AR is still to be clarified.

The dramatic increase in the prevalence of allergic diseases during the past decades is more likely to be the result of changes in environmental factors, accompanied by epigenetic changes in the human genome. By using *Adam33*^−/−^ knock out mouse, a recent report has shown substantial interaction between *Adam33*-mediated airway remodeling and sensitivity to allergen exposure, leading to allergic inflammation and bronchial hyperresponsiveness in early life [37]. The present work is the first study to report the association between methylation level of *ADAM33* and AR risk in terms of the interaction between pet-exposure and *ADAM33* gene promoter methylation. The underlying disease mechanism of this effect remains unknown. However, this study suggests that it is important to examine not only the effect of early-life risk factors, but also their interaction with the DNA methylation level of candidate AR genes.

There are several limitations to our study. First, we used a relatively small sample size, hence there could be a possibility of overestimating the significance of the association of *ADAM33* methylation with AR. Furthermore, the fact that all the children recruited from only one ENT clinic might introduce selection bias into the study. However, we speculate that the relationship between *ADAM33* methylation and pet exposure is involved in AR onset. Second, there were several risk factors that could confound the interaction between pet exposure and the DNA methylation levels of *ADAM33* in children with AR, including disinfection habits of pet owners, mode of delivery, etc. Furthermore, since RNA quality was not good enough for measuring expression level of *ADAM33*, further studies are needed to investigate the potential differential expression pattern of *ADAM33* in AR. To overcome these limitations, a prospective cohort study with bigger sample size will be conducted in the future.

## Conclusions

In conclusion, the present findings suggest that early-life pet exposure is related with higher risk of developing AR, interacting with *ADAM33* methylation level in a cohort of Chinese children. We provide evidence for the important roles of gene-environment interaction in the development of AR.

## Supplementary Information

**Additional file 1: Table S1.** Description of data: twenty-five AR genes retrieved from Phenopedia database and references to the original publication reporting their association to AR.

**Additional file 2: Table S2.** Description of data: Target DNA methylation sequence information.

**Additional file 3: Figure S1.** Description of data: Bisulfite conversion efficiency in PBMC samples from AR patients and controls.

**Additional file 4: Table S3.** Description of data: Different methylation CpG sites between AR patients and healthy controls.

**Additional file 5: Figure S2.** Description of data: Correlation of methylation status of the CpG site at the position of 24 bp of the first amplicon of ACE with eosinophil counts (Spearman’s rank correlation).

## Data Availability

The datasets used and/or analyzed during the current study are available from the corresponding author on reasonable request.

## Abbreviations

AR: Allergic rhinitis
PBMC: Peripheral blood mononuclear cell
GWAS: Genome-wide association studies
SPT: Skin prick test
ROC: Receiver operating characteristic curve
AUC: Area under curve
FDR: False discovery rate
ADAM33: Disintegrin and metalloproteinase 33
