# Supplementary figures and images for: Interaction between early-life pet exposure and methylation pattern of ADAM33 on allergic rhinitis among children aged 3–6 years in China
Source: Allergy Asthma Clin Immunol. 2021 May 1;17:44. doi: 10.1186/s13223-021-00526-5 (PMC8088023; doi:10.1186/s13223-021-00526-5)

Figure S1: Bisulfite conversion efficiency in PBMC samples from AR patients and controls


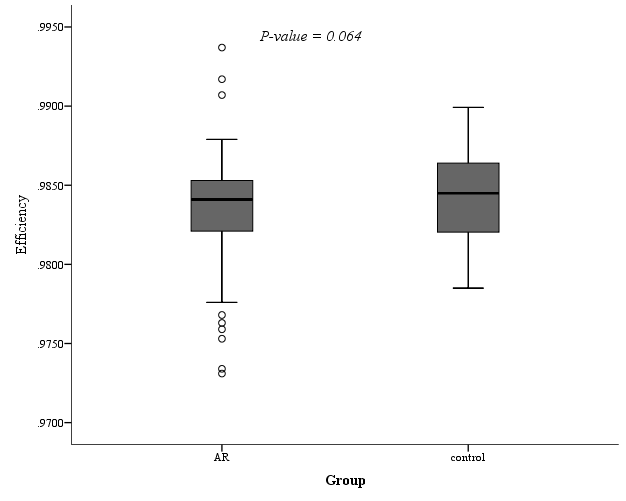

Supplement: Supplementary file 3 — Additional file 3: Figure S1. Description of data: Bisulfite conversion efficiency in PBMC samples from AR patients and controls. [file 13223_2021_526_MOESM3_ESM.docx]
