# Supplementary material for: Interaction between early-life pet exposure and methylation pattern of ADAM33 on allergic rhinitis among children aged 3–6 years in China
Source: Allergy Asthma Clin Immunol. 2021 May 1;17:44. doi: 10.1186/s13223-021-00526-5 (PMC8088023; doi:10.1186/s13223-021-00526-5)

Figure S2: Correlation of the methylation status of the CpG site at the position of 24 bp of the first amplicon of ACE with the total eosinophil count (Spearman’s rank correlation).


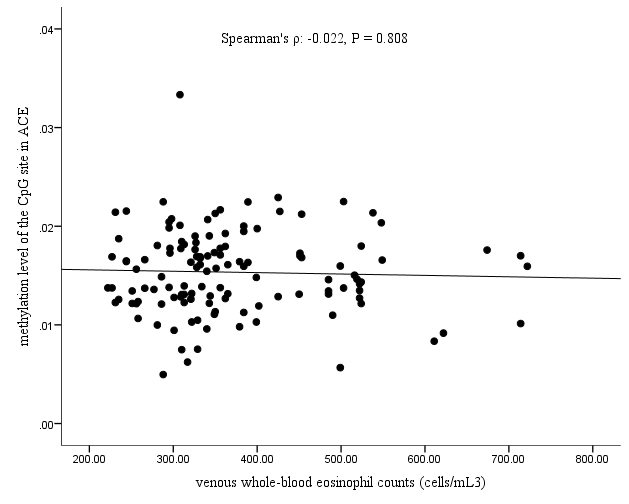

Supplement: Supplementary file 5 — Additional file 5: Figure S2. Description of data: Correlation of methylation status of the CpG site at the position of 24 bp of the first amplicon of ACE with eosinophil counts (Spearman’s rank correlation). [file 13223_2021_526_MOESM5_ESM.docx]
